# Chronic Caloric Restriction and Exercise Improve Metabolic Conditions of Dietary-Induced Obese Mice in Autophagy Correlated Manner without Involving AMPK

**DOI:** 10.1155/2013/852754

**Published:** 2013-05-12

**Authors:** Mingxia Cui, Han Yu, Jinli Wang, Junjie Gao, Ji Li

**Affiliations:** ^1^Department of Pharmacology, Key Laboratory of Preclinical Study for New Drugs of Gansu Province, School of Basic Medical Science, Lanzhou University, Lanzhou 730000, China; ^2^Neuroscience Program, Department of Biochemistry, University at Buffalo, Buffalo, NY 14214, USA; ^3^Department of Pharmacology and Toxicology, University at Buffalo, Buffalo, NY 14214, USA; ^4^Department of Cardiology, Shuguang Hospital Affiliated to Shanghai University of Traditional Chinese Medicine, Shanghai 201203, China

## Abstract

*Aim*. To investigate the role of AMPK activation and autophagy in mediating the beneficial effects of exercise and caloric restriction in obesity. *Methods*. Dietary-induced obesity mice were made and divided into 5 groups; one additional group of normal mice serves as control. Mice in each group received different combinations of interventions including low fat diet, caloric restriction, and exercise. Then their metabolic conditions were assessed by measuring serum glucose and insulin, serum lipids, and liver function. AMPK phosphorylation and autophagy activity were detected by western blotting. *Results*. Obese mice models were successfully induced by high fat diet. Caloric restriction consistently improved the metabolic conditions of the obese mice, and the effects are more prominent than the mice that received only exercise. Also, caloric restriction, exercise, and low fat diet showed a synergistic effect in the improvement of metabolic conditions. Western blotting results showed that this improvement was not related with the activation of AMPK in liver, skeletal muscle, or heart but correlates well with the autophagy activity. *Conclusion*. Caloric restriction has more prominent beneficial effects than exercise in dietary-induced obese mice. These effects are correlated with the autophagy activity and may be independent of AMPK activation.

## 1. Introduction

Caloric restriction (CR) and exercise have been considered to have beneficial effects on human health, including reducing the risks for the development of diabetes, cardiovascular disease, and cancer [1, 2]. Studies have shown that CR and exercise can improve the metabolic conditions in obesity, but the underlying mechanism is still unclear. The AMP-activated protein kinase (AMPK), as an important energy sensor, can be activated by the increased AMP/ATP ratio or ADP in energy deprival state [3]. AMPK activation plays important roles in adjusting the metabolic pathways to restore the ATP level in both short-term and long-term manner [4]. Because of this activity, it has been considered as a potential mediator of the effects of CR and exercise. However, whether AMPK activation is induced in chronic CR or exercise is still an unresolved issue due to distinct observations on the AMPK activity after long-term CR or exercise in mouse [5].

Autophagy is an intracellular recycling pathway that functions during basal conditions but can be induced under stress such as starvation [6]. Recent findings established the relationship between insulin resistance in obesity and the decreased autophagy activity in liver, and restoration of Atg7 was shown to enhance the systemic glucose tolerance in mice [7]. Further, it has been shown that Bcl2-regulated autophagy is indispensable in mediating the improvement of glucose homeostasis by either long- or short-term exercise [8]. Since AMPK has already been known to participate in regulating autophagy activity by activating Ulk1 through direct phosphorylation [9], we wonder if chronic CR and exercise can induce autophagy in obese mice and if the improvement in metabolic conditions is correlated with the autophagy activity. Further, we also sought to investigate if there is any relationship between induced autophagy and AMPK activity in obese mice.

To study the relationship between the chronic CR, exercise, and autophagy and whether AMPK activation is involved in this process, we used high fat diet to induce the obese mice models, and then we investigated the effects of different intervention on the improvement of mice metabolic conditions. Then, we examined the level of LC3 proteins and AMPK phosphorylation to investigate the relationship between metabolic improvement and AMPK activation, as well as the relationship with autophagy activity.

## 2. Materials and Methods

### 2.1. Mice Models

Studies were conducted on 6-week-old male C57 mice, which were fed with high fat diet containing 58% fat (kcal%, total calorie 5.56 kcal/g). Mice in the control group were fed with normal 5% fat diet (kcal%, total calorie 3.4 kcal/g). Six mice, from the experimental and the control group, respectively, were taken and measured for body weight and 24-hour calorie intake every other week.

### 2.2. Intervention of CR and/or Exercise

The first two weeks were adaption period. High fat diet was switched to low fat diet, and the running speed of the exercise groups was 8 m/min, 10 min per day, and 7 days a week. The following 6 weeks were therapeutic period. The calorie intake of CR group is around 70% of that of the model control group, a level that has been reported to effectively protect against high fat diet induced obesity in mice [10]. The running speed was gradually increased from 8 to 22 m/min within one week, 30 min/day, and 5 days/week, as described by Reznick et al., which is considered as sufficient to increase the AMP/ATP level and activate AMPK [11]. The mice were grouped as in Table 1. 

### 2.3. Adipocytokines, Serum Glucose, and Insulin

Serum glucose levels were tested by using Glucose (HK) Assay Kit (Sigma-Aldrich). Adipocytokines ad insulin levels were assessed by ELISA.

### 2.4. Serum Lipids

Triglycerides levels were tested by Triglyceride (GPO Trinder) Reagent A kit. To quantitatively determine the HDL cholesterol in serum, Wako HDL-Cholesterol E assay was used by phosphotungstate-magnesium salt precipitation.

### 2.5. Liver Function

ALT and AST tests kits (American Screening Corporation) were used to evaluate liver function of the mice. Standard HE staining was used to evaluate the morphological changes of livers.

### 2.6. Detection of p-AMPK Phosphorylation and LC3 Activity

Liver, skeletal muscle, and heart homogenates were resolved by SDS-PAGE, and proteins were transferred onto polyvinylidene difluoride membranes. Rabbit polyclonal antibodies against phosphorylated AMPK, total AMPK, and LC3 and anti-rabbit secondary antibodies were purchased from Cell Signaling. LC3-II/LC3-I is used as an index of autophagy activity. 

### 2.7. Statistical Analysis

Values are expressed as means ± SE. Data were analyzed by a two-tailed, independent Student's *t*-test. *P* values less than 0.05 were considered statistically significant.

## 3. Results

### 3.1. Features of Dietary-Induced Obese Mice

#### 3.1.1. Body Weight, Caloric Intake, and Organs Weight

The calorie intake of the DIO mice was consistently higher than that of the control group, and the body weight also increased more rapidly. The weight of control group became stable at week 10, while that of the DIO group continued increasing even at the end of this study. Significant difference exists in the weight, liver, mesenteric fat, postperitoneal fat, and the total visceral fat between the two groups. The weight of the heart also had a significant increase, while no obvious difference was observed in the weight of skeletal muscle. Similarly, the coefficient (organ weight/100 g body weight) of liver, mesenteric fat, postperitoneal fat, and the total visceral fat, increased as well, but that of heart and skeletal muscle had decreased significantly. 

#### 3.1.2. Serum Biochemical Indices

By the end of this study, the DIO mice had exhibited hyperinsulinemia, insulin resistance, and increase in cholesterol, triglycerol, HDL-cholesterol, and LDL-cholesterol. Meanwhile, ALT and AST in the serum also increased. These results showed the obese mice models were successfully induced by using high fat diet.

### 3.2. Effects of Intervention

#### 3.2.1. Body Weight and Caloric Intake

**Figure 1:**
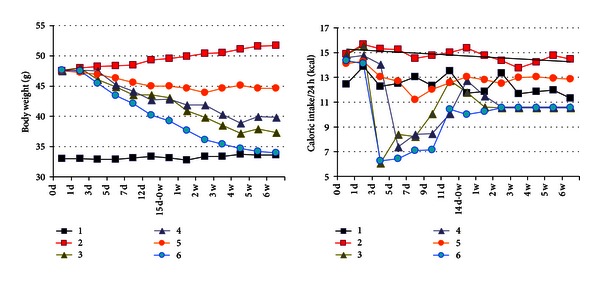
Change in body weight and calorie intake.

The intake of the Groups 3 and 6, whose diet was switched from high fat to low fat, has decreased at the initial stage. After two-week adaption, the intake of these two groups has gradually recovered and got stable. The calorie intake of the exercise group with high fat diet was significantly lower than that of Group 2. At the end of this experiment, the body weights of Groups 3~6 were all significantly lower compared with Group 2 (Figure 1).

#### 3.2.2. Adipocytokines

**Figure 2:**
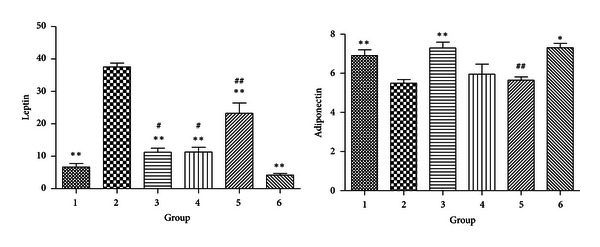
Levels of lectin and adiponectin (*P < 0.05 compared with Group 2. **P < 0.01 compared with Group 2; ^#^ *P* < 0.05 compared with Group 1. ^##^ *P* < 0.01 compared with Group 1).

The level of leptin, which is an appetite-inhibiting adipokine, in DIO mice significantly decreased after CR and/or exercise, and the level of leptin decreased to a similar extent in the CR treated groups regardless of HFD or LFD. Exercise combined with CR and low fat diet further reduced the leptin level. The level of adiponectin was significantly reduced in DIO mice but was recovered in the groups treated with LFD and CR, regardless of exercise. However, in HFD groups neither CR nor exercise had a significant effect on adiponectin level (Figure 2). 

#### 3.2.3. Serum Glucose and Insulin

**Figure 3:**
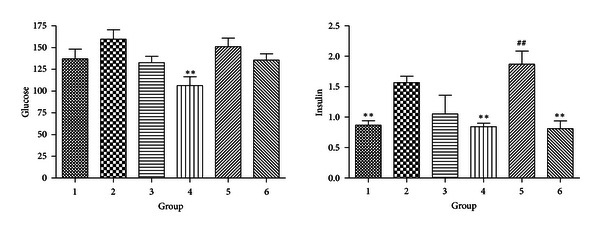
Levels of serum glucose and insulin (*P < 0.05 compared with Group 2. **P < 0.01 compared with Group 2; ^#^ *P* < 0.05 compared with Group 1. ^##^ *P* < 0.01 compared with Group 1).

Serum glucose slightly increased in DIO mice and reduced to a small extent in mice with all the treatments, but most of the differences were not statistically significant. Only the group of HFD plus CR showed a significant improvement. The insulin level also increased in DIO mice, but to a higher degree, suggesting insulin resistance was induced in the obese model and can be recovered by CR or CR plus exercise. Interestingly, however, LFD with CR did not improve the insulin resistance conditions as HFD plus CR did. This plausible result was probably caused by the large variation in this group's insulin level, since the average actually exhibited a decreasing trend when compared with DIO group (Figure 3). 

#### 3.2.4. Serum Lipids

**Figure 4:**
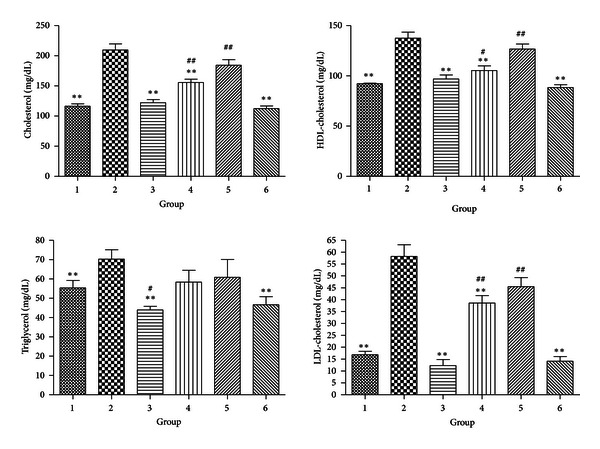
Levels of serum lipids (*P < 0.05 compared with Group 2. **P < 0.01 compared with Group 2; ^#^ *P* < 0.05 compared with Group 1. ^##^ *P* < 0.01 compared with Group 1).

Hyperlipidemia was found in the DIO mice. Among the treatments, CR alone can significantly decrease the cholesterol and LDL-cholesterol levels. Further, when combined with LFD and/or exercise, the effects became even more prominent. LFD and CR can reduce the level of triglyceride either with or without exercise training (Figure 4). 

#### 3.2.5. Liver Function

**Figure 5:**
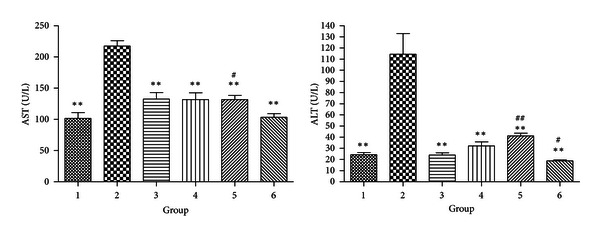
Levels of AST and ALT in serum (*P < 0.05 compared with Group 2. **P < 0.01 compared with Group 2; ^#^ *P* < 0.05 compared with Group 1. ^##^ *P* < 0.01 compared with Group 1).

**Figure 6:**
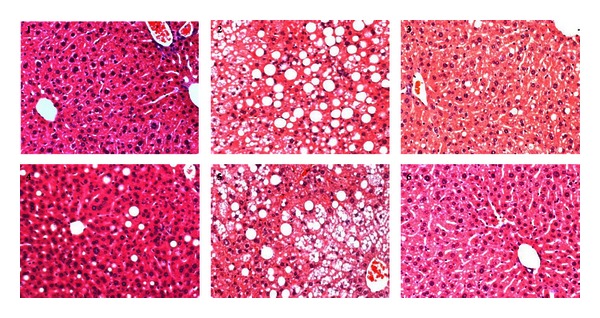
Representative images of H&E staining of liver sections (20x). The numbers on the upper-left corner indicate the group. The liver in Group 1 is generally normal, while obvious hepatic steatosis can be found in Group 2. Hepatic steatosis is much relieved in Group 3, 4, and 6, and that of Group 5 is slightly improved.

Alteration in AST and ALT levels has been shown to be related with metabolic conditions and directly associated with insulin resistance [12]. In addition, these markers can be also used to assess the extents of liver injury in metabolic disorders. Therefore, we further examined their serum levels. While both AST and ALT levels increased in DIO mice, they can be reduced to normal level by either CR or exercise. In consistence with the hyperlipidemia conditions, different extents of hepatic steatosis were found in the HE staining of DIO liver sections. The finding of fatty livers in these mice also explained the increase in serum ASL and ALT levels. As expected, steatosis was obviously improved in the treatment groups, in line with the decrease in serum lipids and transaminase levels (Figures 5 and 6).

#### 3.2.6. Activation of p-AMPK and LC3

**Figure 7:**
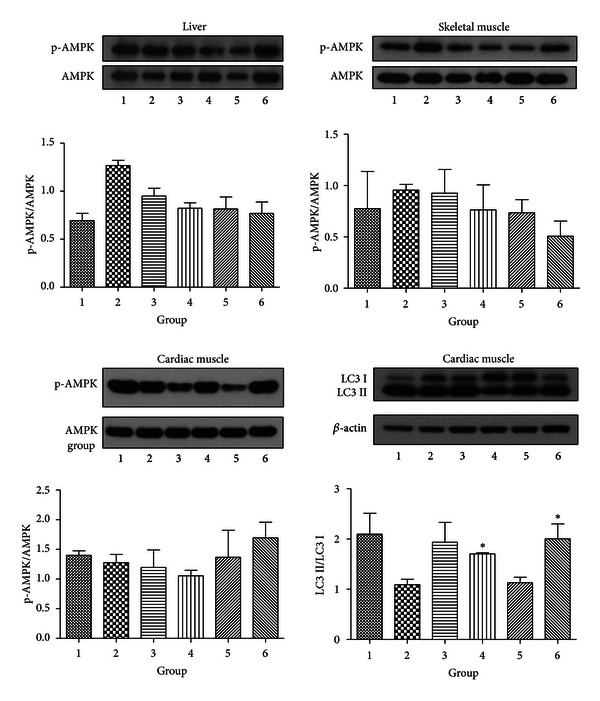
Representative images of the immunoblotting of AMPK in liver, skeletal muscle, cardiac muscle, and LC3 in cardiac muscle. The levels of LC3 II exhibit a significant increase in Groups 4 and 6 compared with that in Group 2, and such trend can also be seen in Group 3. On the other hand, no significant difference in p-AMPK is found in liver, skeletal muscle, or cardiac muscle.

To investigate the relationship between the improvements in metabolic conditions and AMPK activation, we examined the levels of AMPK phosphorylation in liver, skeletal muscle, and cardiac muscle. The results showed that there was no significant difference in AMPK phosphorylation among groups in any of these tissues (Figure 7), implying the AMPK activation does not contribute to the long-term benefits of caloric restriction. Next, we evaluated the autophagy activity in cardiac muscle as represented by LC3 II/LC3 I ratio. The results demonstrated that autophagy activity was significantly elevated in Groups 4 and 6, and such trend was also quite obvious in Group 3 as compared with Group 2 (Figure 7). Considering the facts that these three groups were treated with caloric restriction and showed consistent improvement in metabolic indices, it strongly indicates autophagy activity is responsible for the metabolic improvements in caloric restriction conditions.

## 4. Discussion

The activation of AMPK has been considered as an important factor in adapting the body to the state of energy stress. Upon sensing an increased level of AMP/ATP ratio, AMPK can be fully activated by an increased phosphorylation level at Thr^172^. Activated AMPK can interact with numerous pathways including that of SIRT, mTOR, and autophagy and regulate the activities including mitochondrial biogenesis, glucose uptake, and lipid metabolism [4]. The functions of AMPK imply that its activation may participate in mediating the beneficial effects of chronic caloric restriction and exercise. However, whether AMPK activation plays a part in caloric restriction and exercise is still controversial [5]. In this study, high fat diet was used to induce the obese mice models, and then interventions of CR and exercise were used to improve the metabolic conditions. After that, we examined the level of AMPK phosphorylation in liver, skeletal muscle, and heart tissues to investigate the relationship between metabolic improvement and AMPK activation. 

As expected, two-month feeding of high fat diet successfully induced the obesity and altered the metabolic state of the model mice, which was characterized by increased fat accumulation, insulin resistance, and impaired liver function. However, to most of the indices, exercise alone failed to make a significant improvement, while the effects of caloric restriction were quite prominent. Though it is plausible that exercise alone did not have an obvious effect, which may be caused by an insufficient dosage, it did have a synergistic effect when combined with CR. Moreover, because we are more concerned with the correlation between metabolic improvement and levels of signaling proteins, instead of the effects of individual interventions, this result should not preclude us from getting a reasonable conclusion. Our results indicate that the activation of AMPK is not closely correlated with the improvement of the metabolic conditions of the diet induced obese mice. Thus, the long-term beneficial effects of chronic CR and exercise may not be mediated through AMPK activation.

The result on AMPK in our study is in consistence with that in the study of Gonzalez et al. [13], which showed AMPK activation did not respond to chronic caloric restriction or even fasting in skeletal muscle, cardiac muscle, or liver. However, these results appeared to be contradictory to some other studies, in which CR was shown to lead to AMPK activation [14–16]. In the study of Edwards et al. [14], it was shown that a life-long CR was able to protect the myocardium against ischemia/reperfusion (I/R), and this protective effect was demonstrated to involve AMPK activation, because inhibition of AMPK activation was able to abolish the protection. 

Indeed, AMPK can interact with multiple pathways that may assist in the adjustment of body to different energy metabolic states, and there is also pharmacologic evidence supporting the role of AMPK in mediating CR effects, in which resveratrol was shown to activate AMPK by decreasing ATP production [17]. Therefore, the question why there are different observations concerning AMPK activation in chronic CR and exercise still needs to be clarified. In a previous study, it was proposed that a lack of an effect of CR on AMPK activity is probably due to that the energetic challenge presented by CR was not severe enough to cause the changes in ATP and AMP to activate AMPK. This level of energy challenge brought by CR, however, should be sufficient to affect the metabolic states of mice as shown in both their and this study [13]. Therefore, this may be an explanation on why chronic CR and exercise did not induce the activation of AMPK, and further, it suggests that the beneficial effects of chronic CR and exercise are mediated by some other pathways instead of AMPK activation. 

It is possible that AMPK only becomes activated in more severe and acute energy deprival conditions. For instance, in the study of Edwards et al. [14], a life-long CR could indeed protect myocardium from I/R injuries accompanied by an increase in p-AMPK level, but this does not indicate that AMPK became activated in the basal condition. Also, the cancelling of protection by AraA only proves the AMPK was activated in I/R, which represented an acute and a much more severe energy stress situation than that in chronic CR or exercise. However, the more active AMPK during I/R after a life-long CR does imply a mechanism by which AMPK was sensitized by long-term CR.

If these effects are not mediated by AMPK, then there must be other mechanisms that explain why chronic CR and exercise may improve the metabolic conditions. Recent findings have suggested a relationship between autophagic activity and metabolic conditions. The study of Yang et al. [7] showed that loss of autophagy might be responsible for the insulin resistance in obesity, which was characterized by the downregulation of Atg7, and restoration of Atg7 was able to improve the glucose tolerance in mice. In another research, both long-term and short-term exercises were shown to be able to improve the insulin sensitivity, and this effect depended on induced autophagy [8]. In the light of these findings, we examined the level of conversion of unlipidated LC3 I to LC3 II (autophagosome-membrane-associated lapidated form) in the heart tissues. The results clearly exhibited a trend which is consistent with the change in metabolic conditions in the interventional groups. Specifically, a significant increase in autophagy activity was found in Group 6 mice, and this group also showed a much more prominent trend in increased insulin level, which indicated an improvement in insulin sensitivity. Also, significant increase in the antidiabetic adiponectin, decrease in serum lipids, and improvement in liver function were also exhibited. Therefore, this may be biochemical evidence that an enhanced autophagy activity may participate in regulating the metabolic conditions during chronic CR and exercise, and the effect of autophagy may further extend beyond glucose homeostasis. Moreover, although autophagy in living cells is under control of AMPK, the induction in this context possibly does not depend on AMPK activation.

Since autophagy in living cells is known to be under the regulation of multiple pathways including AMPK, SIRT, and mTOR, it may be important to investigate which among these pathways is responsible for the activation of autophagy during chronic CR and exercise. In the study of He et al., the activation of autophagy in short-term exercise was shown to involve Bcl2-beclin1 complex. Upon dissociating with the antiautophagic Bcl2, beclin1 can activate autophagy, which is mediated via phosphorylating AMPK [8]. However, the long-term exercise might act through a different pathway according to our data, since the p-AMPK level did not change significantly in the groups with long-term exercise. The further study on SIRT and mTOR pathway activity might be helpful to find the molecules responsible for autophagy activation during chronic CR and exercise.

## 5. Conclusions

Our results showed that AMPK activation, which was represented by the level of p-AMPK, did not correlate with the improvement of metabolic conditions in DIO mice, implying AMPK activation may not participate in mediating the beneficial effects of chronic CR or exercise. On the other hand, however, we found the autophagy activity might be related to the improved metabolic conditions but was not correlated with AMPK activation. Thus, we propose that autophagy may play a role in mediating the effects of chronic CR and exercise, but it is not regulated by AMPK activity. However, this hypothesis based on the findings in our research still requires confirmation. For example, AMPK knockout mice might be used to check if these interventions are still effective in the absence of AMPK activation. Finally, detecting SIRT and mTOR pathway activity in the samples may also provide clues on the regulation of autophagy activity in obese mice that received chronic CR or exercise treatment.

## Figures and Tables

**Table 1:** 

Group number	Mice	Treatment
1	Normal	
2	Dietary-induced obesity (DIO)	
3	DIO	Low fat diet with CR
4	DIO	High fat diet with CR
5	DIO	High fat diet with exercise
6	DIO	Low fat diet with CR and exercise
